# Emergence of a daptomycin-non-susceptible *Enterococcus faecium* strain that encodes mutations in DNA repair genes after high-dose daptomycin therapy

**DOI:** 10.1186/s13104-016-2003-9

**Published:** 2016-04-01

**Authors:** Takashi Matono, Kayoko Hayakawa, Risen Hirai, Akira Tanimura, Kei Yamamoto, Yoshihiro Fujiya, Momoko Mawatari, Satoshi Kutsuna, Nozomi Takeshita, Kazuhisa Mezaki, Norio Ohmagari, Tohru Miyoshi-Akiyama

**Affiliations:** Disease Control and Prevention Center, National Center for Global Health and Medicine, 1-21-1 Toyama, Shinjuku-ku, Tokyo, 162-8655 Japan; Department of Hematology, National Center for Global Health and Medicine, Tokyo, Japan; Microbiology Laboratory, National Center for Global Health and Medicine, Tokyo, Japan; Pathogenic Microbe Laboratory, Research Institute, National Center for Global Health and Medicine, Tokyo, Japan

**Keywords:** Daptomycin, *E. faecium*, Whole-genome sequence

## Abstract

**Background:**

An increasing number of reports have documented the emergence of daptomycin-nonsusceptible *Enterococcus* in patients during daptomycin therapy. Even though several mechanisms for daptomycin-nonsusceptibility have been suggested, the potential genetic mutations which might contribute to the daptomycin-nonsusceptibility are not fully understood.

**Case presentation:**

We isolated a vancomycin-susceptible, daptomycin nonsusceptible *Enterococcus faecium* strain from a patient with acute lymphocytic leukemia who received high-dose daptomycin therapy for *E. faecium* endocarditis. Whole-genome sequencing analysis revealed mutations within genes encoding DNA repair proteins MutL and RecJ of the daptomycin-nonsusceptible *Enterococcus* strain which might have facilitated its emergence.

**Conclusions:**

We identified the mutations of DNA mismatch repair genes in a clinical isolate of daptomycin nonsusceptible *E. faecium* which emerged in spite of high-dose daptomycin therapy. The finding implicates the possible association of DNA repair mechanism and daptomycin resistance. Careful monitoring is necessary to avoid the emergence of daptomycin non-susceptible isolates of *E. faecium* and particularly in cases of long-term daptomycin use or in immunocompromised patients.

## Background

Daptomycin (DAP) is a lipopeptide antibiotic that exhibits potent activity against gram-positive bacteria, including vancomycin-resistant enterococci (VRE); however, an increasing number of reports have documented the emergence of daptomycin-nonsusceptible *Enterococcus* (DNSE) in patients during DAP therapy [1–4]. Even though several mechanisms for daptomycin-non-susceptibility have been suggested [5, 6], the potential genetic mutations which might contribute to the daptomycin-nonsusceptibility are not fully understood. In this report, we describe vancomycin-susceptible, daptomycin non-susceptible *Enterococcus* (DNSE) *faecium* strain from a patient with acute lymphocytic leukemia who received high-dose DAP therapy. The whole-genome sequencing analysis revealed mutations within genes encoding DNA repair proteins MutL and RecJ.

## Case presentation

A 32-year-old Japanese man with Philadelphia chromosome-positive acute lymphocytic leukemia (ALL) developed fever during chemotherapy with dasatinib and doxorubicin with dexamethasone for treatment of ALL relapse approximately 3 months after hematopoietic stem cell transplantation. The patient’s blood culture was positive for *E. faecium,* and, as he was allergic to vancomycin, teicoplanin therapy was initiated. Dasatinib and doxorubicin were discontinued immediately. The minimum inhibitory concentrations (MICs) of various antibiotics are listed in Table 1 (EFM01). Although the MIC of teicoplanin for the *E. faecium* strain was ≤2 mcg/ml, and the patient’s serum teicoplanin trough was maintained between 20 and 22 mcg/ml, *E. faecium* was consistently detected in his blood cultures for more than 3 weeks. In addition, the patient was neutropenic during this period, with neutrophil counts between 300 and 830 neutrophils/ml.

**Table 1:** Susceptibility profile of resistance genes of *Enterococcus faecium* isolates

Isolate	Resistance genes						Minimum inhibitory concentrations (MIC) (μg/ml)/Interpretive criteria^a^								
	*Aac(6′)*-*Ii*	*Ant(6)*-*Ia*	*Aph(3′)*-*III*	*ErmB*	*MsrC*	*TetM*	PCG	ABPC	EM	MINO	VCM	TEIC	LVFX	LZD	DAP
EFM01	+	+	+	+	+	+	≥16/R	≥16/R	≥8/R	8/I	1/S	≤2/S	≥8/R	≤2/S	4/S
EFM02	+	+	+	+	+	+	≥16/R	≥16/R	≥8/R	8/I	1/S	≤2/S	≥8/R	≤2/S	256/–

*PCG* penicillin G, *ABPC* ampicillin, *MINO* minocycline, *VCM* vancomycin, *TEIC* teicoplanin, *LVFX* levofloxacin, *LZD* linezolid, *DAP* daptomycin, *S* susceptible, *I* intermediate, *R* resistant

^a^MIC interpretive criteria, per the clinical and laboratory standards institute (CLSI; M100–S24) [20]

After consulting the infectious disease service for recommendations on treating the persistent *E. faecium* infection, the treatment plan was modified to include gentamicin therapy (1.3 mg/kg every 12 h), and imaging studies and an endoscopy were ordered to identify the nidus of the persistent *E. faecium* bacteremia. A transthoracic echocardiogram subsequently revealed a vegetation, measuring a few millimeters in size, on the patient’s aortic valve. Meanwhile, chest and abdominal CT scans detected a thickened colon wall, but no other lesions, and a PET scan failed to identify a potential source of the infection. A colonoscopy, however, revealed erosion throughout the colonic mucosa, which was considered consistent with graft versus host disease and was considered the likely entry site of *E. faecium* into the bloodstream.

As the *E. faecium* bacteremia persisted for 2 weeks after initiation of the gentamicin therapy (gentamicin MIC was 16 mcg/ml) in combination with teicoplanin, the patient was switched to 10 mg kg^−1^ day^−1^ DAP [DAP; Etests indicated that the MIC of DAP for the *E. faecium* strain was 4 mcg/ml as in Table 1 (EFM01)]. After initiation of DAP therapy, the patient’s fever subsided and subsequent blood cultures were negative. As a result, after receiving the initial dose of DAP for 18 days, the dose was reduced to 6 mg kg^−1^ day^−1^. However, 1 day after reducing the dose, the patient developed fever again and his blood culture tested positive for *E. faecium* (DAP MIC, per Etest: 256 mcg/ml). The MICs for other antibiotics are listed in Table 1 (EFM02). DAP was therefore discontinued, and treatment with intravenous linezolid (600 mg every 12 h) was initiated. While blood cultures were negative after 2 days of linezolid therapy, the patient unfortunately passed away owing to exacerbation of the ALL at 26 days after initiation of treatment with linezolid.

Molecular analysis of the daptomycin-susceptible (EFM01; isolated prior to the initiation of DAP) and daptomycin non-susceptible *E. faecium* (DNSE; EFM02) isolates was conducted in the Pathogenic Microbe Laboratory at the Research Institute of the National Center for Global Health and Medicine in Tokyo, Japan. The strains were cultured in brain heart infusion (BHI) broth overnight, and genomic DNA was purified using a DNeasy Blood & Tissue kit (Qiagen, Venlo, Netherlands). The genomes of the two isolates were then subjected to MiSeq sequencing using Nextera XT library kits (Illumina, Inc., San Diego, CA, USA), according to the manufacturer’s instructions. Approximately 1 million pair-end reads (301 base pairs [bp] × 2) were obtained from each genome and analyzed using CLC Genomics Workbench software (CLC bio, Aarhus, Denmark). The reads from each isolate were trimmed by screening for base quality (quality score limit = 0.05; reads that contained greater than two ambiguous nucleotides or that were less than 15 bp in length were removed), and then used to generate de novo genome assemblies, respectively. Meanwhile, the contigs were used as the reference genome. The reads from each isolate were then mapped to the reference genome, and variants were detected using CLC Genomics Workbench program that is based on the algorithm developed by Smith and Waterman (1981) [7]. For these analyses, the following detection parameters were used: 95 % coverage and more than 10 overlapping reads. Because the settings used can yield false-positive variants, each putative variant was manually confirmed by examining the mapping results. The resulting sequencing data were registered with the DNA Data Bank of JAPAN (DDBJ, accession number DRA03513). Furthermore, to annotate variants that were unique to the strains examined in this study, the genome sequence of *E. faecium* Aus0085 was used as a Ref. [8].

The MICs of multiple antimicrobials for the two *E. faecium* isolates, as well as the antimicrobial resistance genes encoded by these organisms, as identified by analysis of contigs using the ResFinder program [4], are summarized in Table 1. Comparison of the EFM01 and EFM02 genomes at SNP level based on whole genome sequencing indicated that EFM02 was derived from EFM01. While EFM02 contained 40 variants that were not present in EFM01, each of the variants identified in EFM01 were present in EFM02. The variants that resulted in amino acid substitutions within the genome of EFM02 compared to that of EFM01 are summarized in Table 2.

**Table 2:** Nonsynonymous nucleotide mutations identified between the daptomycin non-susceptible *Enterococcus faecium* (EFM02) strain and the daptomycin-susceptible *Enterococcus faecium* (EFM01) strain

Genes	*RecJ*	*MutL*	*FusA*	–	*HyuA*	*DapB*	–	*ManX*	*YcaB*	*EbpR*
locus_tag	EFAU085_01331	EFAU085_00136	EFAU085_00055	EFAU085_00149	EFAU085_00226	EFAU085_00409	EFAU085_00744	EFAU085_00813	EFAU085_01095	EFAU085_01417
Predicted gene products	DNA repair protein	DNA mismatch repair protein	Translation elongation factor G	ABC transporter	Hydantoinase/oxoprolinase	Dihydrodipicolinate reductase	Membrane protein	PTS system, Mannose/fructose/sorbose-specific IIAB component	Calcium-translocating P-type ATPase, PMCA-type	M protein trans-acting positive regulator
Predicted amino acid change	Tyr434 Cys	Leu286	Arg626 Cys	Gly190 Asp	Thr570 Ala	Ala190 Val	Phe89 fs	Met1	Ala108 fs	Pro199 Leu
Nucleotide mutation	1301A > G	1876C > T	857T > A	569G > A	1708A > G	569C > T	260delT	1A > G	315delA	596C > T
Previous reports on the same predicted proteins associated with DNSE				[2]				[2, 5, 21]		

Genes	*GlpQ*	–	–	–	*PspC*	–	*AmpC*	*ytpA*	–
locus_tag	EFAU085_01796	EFAU085_01902	EFAU085_01910	EFAU085_0195	EFAU085_02050	EFAU085_02219	EFAU085_02548	EFAU085_02618	EFAU085_02818
Predicted gene products	Glycerophosphodiester phosphodiesterase family protein	Hypothetical protein	Hypothetical protein	HD domain protein	PspC domain protein	Hypothetical protein	Beta-lactamase	Alpha/beta hydrolase family protein	Hypothetical protein
Predicted amino acid change	Ile283Phe	Trp176	Asn125 fs	Trp118 Arg	Lys5 fs	Ser23 fs	Asn265 fs	Glu59Gly	Arg220 fs
Nucleotide mutation	847A > T	527G > A	374delA	352T > C	14delA	68delC	794delA	176A > G	658delA
Previous reports on the same predicted proteins associated with DNSE				[ 5], [21]	[ 2], [5]				

Reference strain: *Enterococcus faecium* Aus0085 [8]

*ABC* ATP-binding cassette, *A* adenine, *Ala* alanine, *Arg* arginine, *Asn* asparagine, *Asp* aspartic acid, *C* cytosine, *Cys* cysteine, *del* deletion, *DNSE* daptomycin non-susceptible *Enterococcus*, *fs* frameshift, *G* guanine, *Glu* glutamic acid, *Gly* glycine, *Ile* isoleucine, *Leu* leucine, *Lys* lysine, *Met* methionine, *Phe* phenylalanine, *PMCA* plasma membrance Ca2 + ATPase, *Pro* proline, *PTS* Phosphotransferase sytem, *psPC* phage shock protein C, *Ser* serine, *Tyr* tyrosine, *T* thymine, *Thr* threonine, *Trp* tryptophan, *Val* valine

Notably, by comparing the genomes of the two *E. faecium* isolates, we detected mutations that were present in the genes encoding the DNA repair proteins MutL (*mutL*) and RecJ (*recJ*) of the DNSE strain, but not the DAP-susceptible parental strain. We, therefore, investigated whether the disruption of these genes affected the frequency of mutations in the *E. faecium* genome. For these analyses, each strain was cultured overnight in 2 ml of BHI broth. The following day, 2 μL of the resulting cultures was used to inoculate 2 ml of BHI broth, respectively. The cultures were again incubated overnight, diluted in fresh broth, and plated on BHI agar. Subsequently, 11 distinct colonies of each strain (EFM01 and EFM02) were harvested, and whole-genome sequencing of these isolates was conducted, as described above. The reads obtained from each isolate were mapped to the respective parental genome and analyzed for the presence of newly acquired variants. Because the settings used can yield false positive variants, any variants that were also present in the parental genome were excluded, and each putative variant was manually confirmed by examining the mapping results.

There was only one newly acquired variant identified after analysis of the genomes of the 11 daptomycin-susceptible *E. faecium* (EFM01) after the bacteria had undergone 9.2 generations. Conversely, analysis of the genomes of the 11 DNSE (EFM02) isolates detected 49 variants after the bacteria had undergone 9.4 generations. These findings indicate that the observed alterations to the *mutL* and *recJ* genes may have resulted in a significant increase in the frequency of mutations in the EFM02 genome.

## Discussion

In this report, we characterized a strain of *E. faecium* with high level of DAP resistance (MIC = 256 mcg/ml) that was isolated from a patient with ALL following 20 days of exposure to high-dose DAP (10 mg kg^−1^ day^−1^) for treatment of *E. faecium* endocarditis. Subsequent genomic analyses indicated that this DNSE strain contained mutations within the known DNA repair genes *mutL* and *recJ*, which may have contributed to the acquisition of DAP resistance. Although dasatinib was reported to have effect on DNA repair pathways in human cancer cell lines [9], the association of DNA repair gene mutations of bacterial isolates with dasatinib or doxorubicin has not been reported to the best of our knowledge. In this case, dasatinib and doxorubicin were discontinued at the time of the first episode of *E. faecium* bacteremia, and thus, the patient was not receiving these drugs during DNSE emergence.

In a previous study of 42 cases of DNSE infection, which included five cases due to vancomycin-susceptible DNSE, only two VRE strains (4.2 %) exhibited DAP MICs ≥128 mcg/ml [10]. Meanwhile, the most common underlying disease associated with DNSE infection was hematologic malignancy (35 %), which was also present in the current case [10]. Indeed, immunosuppression and prior exposure to cephalosporins and metronidazole are considered independent predictors of infections caused by DNSE [11]. While in vitro analyses indicated that the acquisition of DAP resistance requires at least 6 days of exposure to DAP [6], the median duration of DAP exposure in previous case series of DNSE was 16–19 days [12, 13], which is similar to the duration of DAP treatment in the current case (20 days).

In recent reports of DNSE that developed during DAP therapy, patients received 6 mg/kg DAP [1–3, 14]. Meanwhile, separate studies demonstrated that ≥8 mg kg^−1^ day^−1^ of DAP resulted in improved clinical outcomes in cases of VRE blood stream infections, but that an even higher dose of DAP (≥10 mg kg^−1^ day^−1^) might be required to prevent the development of DAP resistance [6, 15]. In the current case, however, DNSE survived high-dose DAP therapy (10 mg kg^−1^ day^−1^). While recent studies suggest that increases in DAP MICs are associated with decreases in the MIC of beta-lactams [16], further investigation is required to assess whether the inclusion of beta-lactams such as ampicillin might help prevent the development of DNSE. Furthermore, a recent meta-analysis indicated that linezolid is more effective than is DAP for treatment of VRE bacteremia and that linezolid was associated with decreased rates of mortality [17]. However, the side effects associated with this antimicrobial, particularly adverse hematologic reactions, hinder its long-term use.

The mechanisms underlying DAP non-susceptibility in enterococci are not fully understood, but recent reports suggest the involvement of the cardiolipin synthase enzyme as well as several genetic pathways, including those associated with cell membrane phospholipid metabolism and the response of the bacterial cell-envelope to antibiotics [5]. We did not identify mutations that have been previously determined to confer daptomycin non-susceptibility in *E. faecium*, such as *liaFSR*, *yycFGHIJ*, cardiolipin synthetase, or *ezrA* [5]. However, we identified multiple amino acid changes predicting gene products which were reported previously in daptomycin non-susceptibile *E. faecium* isolates by whole-genome analyses, as shown in Table 2. In addition, we identified mutations within the DNA repair genes *mutL* and *recJ* that were unique to the DNSE strain and demonstrated that these mutations might have facilitated the emergence of spontaneous mutations during subculturing. We propose that this increased frequency of mutation might have led to the observed emergence of the DNSE phenotype. Contrary to this hypothesis, in a previous study, Willems et al. failed to detect demonstrable hypermutator phenotypes in oxazolidinone-resistant or -susceptible *E. faecium* isolates with mutations in the *mutLS* locus [18]. However, it is possible that the distinct phenotypes associated with alterations in the *mutL* gene could be due to differences in the genetic position of the individual *mutL* mutations [18]. Meanwhile, the *recJ* gene encodes a 5′-3′ single-stranded DNA-specific exonuclease that was reported to be associated with illegitimate recombination [19]. To the best of our knowledge, there have been no reports that have examined mutations in the *recJ* gene or the role of this protein in *Enterococcus* spp. Further studies are therefore are needed to reveal the association, if any, between mutations in *recJ* and the development of DNSE.

## Conclusions

In conclusion, we isolated a strain of vancomycin-susceptible, DAP non-susceptible *E. faecium*, which survived exposure to high-dose (10 mg kg^−1^ day^−1^) DAP for treatment of *E. faecium* endocarditis. Whole-genome sequencing revealed mutations within the *mutL* and *recJ* genes of the DNSE strain, while in vitro analyses demonstrated that the DNSE strain exhibited higher rates of spontaneous mutation than did the parental strain. Our findings demonstrate that careful monitoring is necessary to avoid the emergence of DAP non-susceptible isolates of *E. faecium*, in spite of high-dose therapy, and particularly in cases of long-term DAP use or in immunocompromised patients such as those with hematological malignancy. Our study did not include the demonstration of the relationship of these DNA repair genes mutations with phenotypic changes, and we were unable to determine the exact mechanism of resistance. Further investigation is necessary to elucidate the mechanism by which *E. faecium* acquires DAP resistance, as well as the contribution of mutations in the DNA mismatch repair genes *mutL* and *recJ* to this process.

## Abbreviations

DAP: daptomycin
RE: vancomycin-resistant enterococci
DNSE: daptomycin-nonsusceptible *Enterococcus*
ALL: acute lymphocytic leukemia
MIC: minimum inhibitory concentration
BHI: brain heart infusion
